# SDFnT-Based Parameter Estimation for OFDM Radar Systems with Intercarrier Interference

**DOI:** 10.3390/s23010147

**Published:** 2022-12-23

**Authors:** Jingqi Wang, Pingping Wang, Ruoyu Zhang, Wen Wu

**Affiliations:** School of Electronic and Optical Engineering, Nanjing University of Science and Technology, Nanjing 210094, China

**Keywords:** intercarrier interference (ICI), orthogonal frequency division multiplexing (OFDM), radar, scale discrete Fresnel transform (SDFnT)

## Abstract

The orthogonal frequency division multiplexing (OFDM) radar suffers from severe performance degradation in range-velocity estimation in high mobility scenarios. In this paper, a novel intercarrier interference (ICI)-free parameter estimation method for OFDM radar is proposed. By employing a scale discrete Fresnel transform (SDFnT), the OFDM radar signals are converted to the scale Fresnel domain, and the orthogonality of subcarriers can be recovered with the optimal scale factor. Furthermore, due to the compatibility of the SDFnT and the discrete Fourier Transform (DFT), the proposed method has low computational complexity and high feasibility for OFDM radar implementation. Simulation results show that the proposed SDFnT-based scheme effectively eliminates the ICI effect for single and multiple targets and achieves high accuracy delay-Doppler estimation for OFDM radar systems in circumstances of high velocity and low SNR with consistency and robustness.

## 1. Introduction

In recent years, multicarrier waveforms have risen in popularity for radar detection due to the advantages of waveform diversity, detection performance, and ease of implementation [1,2,3,4]. Because of its potential in joint radar-communications (JRC) systems, orthogonal frequency-division multiplexing (OFDM), a widely employed multicarrier waveform in communication systems for decades, has attracted considerable interest as a radar waveform [5,6]. However, the orthogonality of OFDM radar subcarriers, like that of OFDM communications systems, is susceptible to Doppler-induced phase changes. As a result, intercarrier interference (ICI), which caused by the loss of the subcarriers’ orthogonality, will significantly degrade the performance of OFDM radar detection in high-mobility applications that have become increasingly common in recent years and may in the future, such as high-speed trains (HST), unmanned aerial vehicles (UAV), vehicle-to-everything (V2X) in smart cities, etc. [7,8,9].

The term “ICI” in the OFDM communication system refers to temporal variations in the channels during one OFDM symbol interval that break the orthogonality of the individual subcarriers and cause power leakage [10]. To estimate and equalize the quickly time-varying channels, numerous ICI mitigations, such as piecewise linear approximation [11], blind frequency offset estimation [12], and windowing [13], have been proposed. Unfortunately, these methods that are designed for communication systems did not construct the system models required for radar signal processing and hence cannot be applied directly to OFDM radar systems.

For radar systems, Doppler effects caused by Doppler difference between subcarriers, which can be interpreted as target range migration or range walk, are well known to degrade the performance of high-speed target detection. A number of compensation approaches for the range migration have been reported, which can be broadly classified as parameter searching techniques with time axis rescaling [14,15,16] and echo autocorrelation techniques with low computational complexity [17,18,19]. Moreover, for OFDM radar systems, many range migration compensation methods have been proposed, including scaling technique [20], frequency hopping schemes [21,22], and special OFDM sequences, such as cyclic shifting sequences [23], phase coding of the same sequence [24], and M-PSK modulation of symbol in pulse compression [25]. However, the Doppler-induced linear phase shift on each sample of the time domain, which is a major source of ICI effects in OFDM radar, was not taken into account in these methods and consequently results in performance degradation when the target velocity is no longer significantly smaller than the maximum unambiguity velocity.

In low-mobility scenarios, it is common to assume that the Doppler frequency shift is considerably smaller than the subcarrier spacing, resulting in a tolerable Doppler-induced phase shift, as in the classical OFDM radar signal processing scheme [26], which estimates target range and velocity using an inverse discrete Fourier transform (IDFT) and a discrete Fourier Transform (DFT) along the delay and Doppler dimensions, respectively. However, in high-mobility conditions, the linear phase shift along the slow-time axis, i.e., the ICI-induced Doppler shift, corrupts the orthogonality between range-induced phase shift and velocity-induced phase shift and therefore is no longer eligible. In order to avoid the performance degradation caused by ICI effects in high-mobility OFDM radar estimation, various ICI mitigation approaches have been proposed by loading OFDM sequences with different constraints, such as repeated symbols [27], good correlation properties [28], and a rank-one symbol matrix [29]. However, due to the restrictions these methods place on transmitted data sequences, OFDM radar waveform optimization and radar-communications integration are particularly challenging. Recently, an alternating projection maximum likelihood (AP-ML) method has been proposed to support ICI mitigation with arbitrary transmit sequences [30]. The authors of [31] formulated the radar delay-Doppler estimation as a joint carrier frequency offset (CFO) and channel estimation problem. These ICI mitigation approaches have been proven to be successful for OFDM radar systems with arbitrary sequences, but they are computationally demanding and difficult to implement.

This paper proposes an ICI mitigation parameter estimation method for OFDM radar systems based on a novel scale discrete Fresnel transform (SDFnT), whose kernel function is a set of orthogonal chirp signals with a tunable chirp rate, which to the best of the authors’ knowledge has never been addressed to eliminate the ICI effect in the literature. The key idea of the SDFnT-based ICI mitigation is to convert the OFDM radar signals to the scale Fresnel domain instead of the frequency domain, resulting in a set of variable chirp sequences according to the scale value. With the optimum scale, the orthogonality of subcarriers can be recovered, and the ICI influence caused by the high-speed target can be effectively eliminated. The proposed method imposes no constraints on the data sequences transmitted by the OFDM radar system, which differs from the works in [27,28,29], opening up a wider range of applications. Furthermore, since the proposed SDFnT and DFT are compatible and computationally equivalent, the SDFnT-based ICI mitigation method has significantly less computational cost than the parameter estimation techniques in [30,31] and can be successfully implemented in current OFDM radar systems. The estimation accuracy of the proposed method is evaluated and compared with the conventional OFDM radar parameter estimation method in [26] in terms of signal-to-noise ratio (SNR) and velocity.

The main contributions can be summarized as follows:

- We propose a novel scale discrete Fresnel transform that can convert a time-domain signal into the scale Fresnel domain, with the resultant projection varying with the value of the non-zero scale factor. With its help, we are able to address the ICI mitigation issue for OFDM radar systems;
- Based on the SDFnT, we develop a brand-new ICI-free parameter estimation method for OFDM radars in high-mobility scenarios. We transfer the received and transmitted OFDM signals to the scale Fresnel domain and convert the ICI-induced phase rotation matrix into an identity matrix by using the optimal value of the scale factor. This approach can effectively eliminate the ICI effect caused by high velocity with low computational complexity;
- We establish a thorough processing flow to ensure that the appropriate scale factor value is obtained and the proposed algorithm can be applied properly regardless of the presence or absence of sufficient prior information;
- We compare the proposed method with the conventional estimation method via extensive simulations and validate the superiority of the proposed method in terms of velocity and SNR, as well as its robustness against the scale factor error.

The remainder of this paper is organized as follows. Section 2 describes the signal models of the OFDM radar with intercarrier interference and discusses the invalidation of conventional parameter estimation. Section 3 proposes the discrete Fresnel transform and then develops an ICI mitigation method based on it using the concise matrix-form representation of the formula derivation. A processing flow of the proposed algorithm is given, including two schemes for the scale factor optimum value calculation with and without enough prior information. Simulation results are given in Section 4. Finally, this paper is concluded in Section 5.

Notations: uppercase bold letters denote vectors and matrices. “∥·∥  and ${\left(\cdot \right)}^{H}$ represent the Euclidean norm and complex conjugate transpose operator, respectively. ${\left(A\right)}_{m,n}$ stands as the matrix element of the column *n* and row *m*. $\mathrm{diag}\left(a_{1},a_{2},\ldots ,a_{n}\right)$ represents the diagonal matrix whose diagonal entries are *a*_1_, *a*_2_,…, *a_n_*.

## 2. Signal Model

Analytically, the transmitted baseband OFDM radar signal *x*(*t*) with *N* subcarriers and *M* symbols can be expressed as:

(1) $$x(t)=\frac{1}{\sqrt{N}}\overset{M-1}{\underset{m=0}{\sum }}\overset{N-1}{\underset{n=0}{\sum }}d_{m}\left(n\right)e^{j2\pi f_{n}t}\mathrm{rect}\left(\frac{t-mT}{T}\right),$$

where dm(n) is the data sequences on the *n*th OFDM subcarrier of the *m*th symbol, $f_{n}=n∆f$ is the frequency of the *n*th subcarrier, ∆f=1/T is the subcarrier spacing, and *T* is the OFDM symbol duration.

The received echo signal of a point target at a distance *R* with a moving velocity *V* can be given by

(2) $$y(t)=\frac{1}{\sqrt{N}}\overset{M-1}{\underset{m=0}{\sum }}\overset{N-1}{\underset{n=0}{\sum }}A_{m}\left(n\right)d_{m}\left(n\right)e^{j2\pi f_{n}\left(t-\tau \right)}e^{j2\pi f_{d}t}\mathrm{rect}\left(\frac{t-mT-\tau }{T}\right),$$

where $A_{m}\left(n\right)$ is the complex factor that describes the phase shift and attenuation and is assumed to be constant, $A_{m}\left(n\right)=A$ hereinafter $f_{d}{=2\mathrm{Vf}}_{c}/c_{0}$ is the Doppler shift, $\tau =2R/c_{0}$ is the time delay, and *c*_0_ is the speed of light.

Sampling *y*(*t*) at t=mT+kt/N for *k* = 0,…, *N* − 1, the discrete time-domain received signal of the *m*th symbol can be written as

(3) $$y_{m}(k)=\frac{1}{\sqrt{N}}\overset{N-1}{\underset{n=0}{\sum }}Ad_{m}\left(n\right)e^{\frac{j2\pi nk}{N}}e^{-j2\pi n\Delta f\tau }e^{j2\pi f_{d}mT}e^{\frac{j2\pi f_{d}kT}{N}}.$$

For a more concise representation of the signal, the received radar observations in (3) can be expressed as

(4) $$Y=A\psi_{f_{d}}F^{H}\gamma_{\tau }D\varphi_{f_{d}},$$

where $Y\in {\mathbb{C}}^{N\times M}$ and $D\in {\mathbb{C}}^{N\times M}$ denote the received echo and the transmitted arbitrary data, in which every column represents one OFDM symbol, and every row represents one time sample and one subcarrier, respectively. $F\in {\mathbb{C}}^{N\times N}$ is the DFT matrix with the element of the *n*th row and the *k*th column as ${\left(F\right)}_{n,k}=1/\sqrt{N}\exp\left(-j2\pi \mathrm{nk}/N\right)$, and

(5) $$\psi_{f_{d}}≜\mathrm{diag}\left(1,e^{j2\pi f_{d}T/N},...,e^{j2\pi \left(N-1\right)f_{d}T/N}\right)\in {\mathbb{C}}^{N\times N}\mathbb{C},$$

(6) $$\gamma_{\tau }≜\mathrm{diag}\left(1,e^{-j2\pi \Delta f\tau },...,e^{-j2\pi \left(N-1\right)\Delta f\tau }\right)\in {\mathbb{C}}^{N\times N},$$

(7) $$\varphi_{f_{d}}≜\mathrm{diag}\left(1,e^{j2\pi f_{d}T},...,e^{j2\pi f_{d}\left(M-1\right)T}\right)\in {\mathbb{C}}^{M\times M}.$$

Clearly, **γ**_τ_ and $\varphi_{f_{d}}$ represent the range-introduced linear phase shift along the frequency axis and the Doppler-introduced linear phase shift along the fast-time axis, respectively. Additionally, $\psi_{\mathrm{fd}}$ in Equation (5) is denoted the Doppler-introduced linear phase shift along the slow-time axis, which is a major source of ICI effects in OFDM radar and results in performance degradation of parameter estimation approaches when the target velocity is no longer significantly smaller than the maximum unambiguity velocity.

Under the assumption that the low-velocity target that satisfies the constraint $f_{d}\ll ∆f$, $\psi_{\mathrm{fd}}$ becomes an identity matrix, the intercarrier interference in the fast-time domain is negligible. Hence, after a DFT transform and an element-wise complex division for the removal of the arbitrary data matrix D, the delay and Doppler shift introduced by the moving target lead to mutually orthogonal phase shifts along the frequency axis and the slow-time axis and can be easily obtained using IDFT and DFT across the columns and rows, respectively [32]. However, in high-mobility scenarios, such as a moving target approaching its maximum unambiguous velocity, the ICI effect is no longer ignorable. In this case, the diagonal matrix $\psi_{\mathrm{fd}}\;$ that represents the ICI-dependent phase shift becomes a non-diagonal matrix after the DFT transform. It breaks the orthogonality among the subcarriers of OFDM radar signals, causing the range-induced phase shift along the frequency axis and the velocity-induced phase shift along the time axis to couple, and consequently causes severe performance degradation of many conventional parameter estimation approaches that do not take into account the ICI effect. Clearly, effective ICI mitigation is important for accurate range-velocity estimation in high-mobility OFDM radar applications.

## 3. SDFnT-Based ICI Mitigation Method

In this section, first, a scale discrete Fresnel transform derived from the discrete Fresnel transform, an integral transformation that comes from classical optics [32,33,34], is proposed, and then a novel OFDM radar range-velocity estimation method based on SDFnT is presented to reduce the ICI effect in high-mobility scenarios.

### 3.1. SDFnT

The discrete Fresnel transform (DFnT) is the discrete form of the Fresnel transform, and the (*l*, *k*)th entry of the *N* by *N* DFnT matrix **Ξ** is defined as

(8) $${\left(\Xi \right)}_{l,k}=\frac{1}{\sqrt{N}}e^{-j\frac{\pi }{4}}\left\{\begin{array}{c} e^{j\frac{\pi }{N}{\left(l-k\right)}^{2}}\;\;N\equiv 0(\mathrm{mod}2)\\ e^{j\frac{\pi }{N}{\left(l+\frac{1}{2}-k\right)}^{2}}\;N\equiv 1(\mathrm{mod}2) \end{array}\right..$$

The DFnT can be used to transform the time-domain signals into the Fresnel domain and can be implemented by the DFT in three steps, making it popular for OFDM radar and communication applications.

In this paper, we present an augmented transform of DFnT, namely the scale discrete Fresnel transform, by introducing a non-zero scale transform factor *α* and making the chirp rate of the SDFnT kernel function adjustable. The (*l*, *k*)th element of the *N* by *N* SDFnT matrix **G** is defined as

(9) $${\left(G\right)}_{l,k}=\frac{1}{\sqrt{N}}e^{-j\frac{\pi }{4}}\left\{\begin{array}{c} e^{j\frac{\pi }{N}{\left(l\alpha -k/\alpha \right)}^{2}}\;\;N\equiv 0(\mathrm{mod}2)\\ e^{j\frac{\pi }{N}{\left(l\alpha +\frac{1}{2}-k/\alpha \right)}^{2}}\;N\equiv 1(\mathrm{mod}2) \end{array}\right..$$

Clearly, the kernel functions of the proposed SDFnT are a set of orthogonal chirp signals with the adjustable chirp rate *N*/(*αT*)^2^. When the scale factor equals one, the proposed SDFnT degenerates to the classic DFnT in Equation (8).

By exploiting the SDFnT, we can convert a discrete signal *x*(*k*) into a sequence in the scale Fresnel domain. The converted signal on the *l*th chirp is given by

(10) $$X\left(l\right)=\left\{\begin{array}{c} \frac{1}{\sqrt{N}}\overset{N-1}{\underset{k=0}{\sum }}x\left(k\right)e^{-j\frac{\pi }{4}}e^{j\frac{\pi }{N}{\left(l\alpha -k/\alpha \right)}^{2}}\;\;N\equiv 0(\mathrm{mod}2)\\ \frac{1}{\sqrt{N}}\overset{N-1}{\underset{k=0}{\sum }}x\left(k\right)e^{-j\frac{\pi }{4}}e^{j\frac{\pi }{N}{\left(l\alpha +\frac{1}{2}-k/\alpha \right)}^{2}}\;N\equiv 1(\mathrm{mod}2) \end{array}\right..$$

In order to comply with the practical OFDM system, *N* is set to be an even number, and we can thus simplify the definition of the SDFnT matrix as $G\in {\mathbb{C}}^{N\times N}$, with elements ${\left(G\right)}_{l,k}=1/\sqrt{N}\exp\left(-j\pi /4\right)\exp\left(j\pi {\left(l\alpha -k/\alpha \right)}^{2}/N\right)$.

It can be readily proved that the SDFnT can be implemented by multiplying additional quadratic phase matrices and the DFT matrix **F**, i.e.,

(11) $$G=\Theta_{1}F\Theta_{2},$$

where **Θ**_1_ and **Θ**_2_ are diagonal matrices whose diagonal entries are ${\left(\Theta_{1}\right)}_{l,l}=\exp\left(-j\pi /4\right)\exp\left({j\pi l}^{2}\alpha^{2}/N\right)$ and ${\left(\Theta_{2}\right)}_{k,k}=\exp\left({j\pi k}^{2}/\left(\alpha^{2}N\right)\right)$, respectively. The representation in Equation (11) makes apparent that the SDFnT has a computational complexity of O(*N*log*N*), which is the same as for DFT processing.

The proposed transform can be considered a projecting process that maps a given signal on the time axis to the scale Fresnel axis. The resulting projection varies with the value of the scale transform factor α, and therefore can be used to re-concentrate the ICI-induced spreading energy.

### 3.2. ICI Mitigation

By performing an *N*-point SDFnT, the transmitted signal **X** and the received signal **Y** can be converted into the scale Fresnel domain, which are given by

(12) $$GX=GF^{H}D$$

(13) $$GY=AG\psi_{f_{d}}F^{H}\gamma_{\tau }D\varphi_{f_{d}}$$

Note that the multiplication of the exponential terms forming the **γ**_τ_ matrix by the columns of the IDFT matrix is equivalent to the cyclic shift of the rows of **F**^H^ downward by ε=NΔfτ, therefore we have

(14) $$F^{H}\gamma_{\tau }=C_{\varepsilon }F^{H},$$

where **C***ε* is the identity matrix that is cyclically shifted downward by *ε*.

The substitution of Equation (14) into Equation (13) yields

(15) $$GY=AG\psi_{f_{d}}C_{\varepsilon }F^{H}D\varphi_{f_{d}}.$$

Similarly, the multiplication of ${G\psi }_{\mathrm{fd}}$ and **C***ε* is equivalent to the cyclic shift of the columns of ${G\psi }_{\mathrm{fd}}\;$ leftward by *ε* columns. Thus, we obtain

(16) $$G\psi_{f_{d}}C_{\varepsilon }=A^{\prime }\gamma_{\tau }G\Gamma ,$$

where $A^{\prime }=\exp\left(j\pi \left(\varepsilon^{2}/\alpha^{2}{+2\varepsilon f}_{d}T\right)/N\right)$ is a scalar related to the target’s range and velocity, and

(17) $$\Gamma ≜\mathrm{diag}\left(1,e^{j2\pi \left(\varepsilon /\alpha^{2}+f_{d}T\right)/N},\cdots ,e^{j2\pi \left(\varepsilon /\alpha^{2}+f_{d}T\right)\left(N-1\right)/N}\right)\in {\mathbb{C}}^{N\times N},$$

is a diagonal matrix that contains all the ICI-dependent phase shifts.

By substituting Equation (16) back into Equation (15), the received signal in the scale Fresnel domain is equivalent to the following

(18) $$GY=A^{\prime \prime }\gamma_{\tau }G\Gamma F^{H}D\varphi_{f_{d}},$$

where A″=A⋅A′ is a constant term associated with *R* and *V*.

In Equation (18), **γ**_τ_ and $\varphi_{f_{d}}$ represent the range introduced linear phase shift along the scale Fresnel axis and the velocity introduced linear phase shift along the fast-time axis, respectively. More importantly, instead of being included in the matrix, $\psi_{\mathrm{fd}}$, in Equation (4) as a linear phase shift on each sample of the time domain, all ICI-induced Doppler effects are evidently incorporated in the matrix **Г** as a linear phase shift on every subcarrier of the scale Fresnel domain after the SDFnT.

From the expression for matrix **Г**, we can see that the projections of the phase change due to the ICI effect on the scale Fresnel axis, vary with the scale transform factor. By changing the value of the scale factor *α*, the projections can be changed, and the ICI effects could be eliminated. When an appropriate value of *α* is chosen, the ICI-induced phase shifts on each subcarrier of the scale Fresnel domain would become one, and the range-induced phase shift along the scale Fresnel axis and the velocity-induced phase shift along the time axis are completely decoupled.

In that case, the matrix **Г** becomes an identity matrix, and the *k*th element of the matrix **Г** is

(19) $$e^{j2\pi \left(\varepsilon /\alpha^{2}+f_{d}T\right)k/N}=e^{j2l\pi }.$$

Then, we can obtain the expression of the scale factor as

(20) $$\alpha =\pm \sqrt{\frac{\varepsilon }{lN-f_{d}T}},$$

where *l* is any integer. For computational convenience, *l* is set to be 0 hereinafter.

Considering ε=NΔfτ and $f_{d}=2Vf_{c}/c_{0}$, we have the optimal value of the scale factor *α*:

(21) $$\alpha_{\mathrm{opt}}=\pm j\sqrt{\frac{NR}{Vf_{c}T^{2}}}.$$

Plug the optimal α_opt_ back into Equation (18), and the ICI-free received signal in the scale Fresnel domain can be written as

(22) $$GY=A^{\prime \prime }\gamma_{\tau }GF^{H}D\varphi_{f_{d}},$$

where A″ is a constant term associated with *R* and *V*.

Clearly, without the ICI-induced Doppler effects, we can easily remove the arbitrary data sequences by performing an element-wise complex division of Equation (12) and Equation (22) and obtaining the phase matrix **P** that carries the range and velocity information along its columns and rows, respectively,

(23) $${\left(P\right)}_{l,m}=\frac{{\left(GY\right)}_{l,m}}{{\left(GX\right)}_{l,m}}.$$

The ICI-free range and velocity estimations for high mobility applications can be obtained by calculating the discrete Fourier transform of every row of **P** and then the inverse discrete Fourier transform for every column of the resulting matrix.

(24) $$Z=FPF^{H}.$$

The resulting matrix **Z** directly represents a two-dimensional radar image in terms of range and Doppler. By finding the peak of **Z**, we can obtain the range *R* and velocity *V* of the high-speed target. The proposed SDFnT-based ICI-free parameter estimation for OFDM radar in high-mobility scenarios is briefly summarized in Algorithm 1.

**Algorithm 1** Proposed SDFnT-based parameter estimation algorithm for OFDM radar **Input**: The transmitted and received time domain signals, **X**, **Y**, and the scale factor    α; **Output**: **Z**, *R*, and *V*; **Step 1:** Convert the transmitted and received signal into the scale Fresnel domain,    **G***^α^***X** and **G***^α^***Y**, via the SDFnT matrix **G***^α^*; **Step 2:** Perform an element-wise complex division on **G***^α^***Y** and **G***^α^***X** to calculate the    phase matrix **P** using Equation (23); **Step 3:** Perform 2D-FFT on matrix **P** to obtain the range-Doppler radar image **Z** by    using Equation (24); **Step 4:** Find the peak of radar image **Z**, and obtain the estimates of range and    velocity, *R* and *V*.

It is obvious that one of the most significant advantages of the SDFnT-based ICI mitigation method is its low computational complexity. In addition, the FFT and IFFT calculations and the computational time of the proposed algorithm are mainly spent on the SDFnT processing, which has approximately the same complexity as FFT. Considering that the sizes of the transmitted and received radar signal matrices are *N* × *M*, the SDFnT-based method requires O(2*MN* + *MN*(log(*N*))) calculations for the SDFnT transform, O(*MN*) for element-wise division, and O(*MN*(log(*N*))) and O(*MN*(log(*M*))) for IFFT across column and FFT across row, respectively. In total, the computational complexity of the proposed approach is O(3*MN* + 2*MN*(log(*N*)) + *MN*(log(*M*))).

It is worth noting that α_opt_ in Equation (21) highly depends on the range and velocity of the target. In some circumstances, we have enough prior information to calculate the optimal value of the scale factor. For example, in the *q*th detection of a tracking radar, the range and velocity of the target have already been known from the last detection, and we can use the (*q* − 1)th estimates of *R* and *V*, i.e., $R^{q-1}$ and $V^{q-1}$, instead of the true values of *R* and *V*, to calculate the α_opt_ using Equation (21).

However, in some other cases, there is no sufficient prior information to determine the optimal α_opt_. For instance, when the radar system first starts up, the range and velocity of the target are unknown, and it is impossible to calculate the true optimal value of α. In this case, the searching method can be employed to yield the best estimation for α.

The maximum range *R*_max_, range resolution ∆*R*, maximum velocity *V*_max_, and velocity resolution ∆*V* can be calculated using the known OFDM radar system parameters, including frequency, bandwidth, number of subcarriers, and symbols. The maximum and minimum values of the scale factor amplitude are defined, respectively, as

(25) $${\left\|\alpha \right\|}_{\max}=\sqrt{\frac{NR_{\max}}{\Delta Vf_{c}T^{2}}},$$

(26) $${\left\|\alpha \right\|}_{\min}=\sqrt{\frac{N\Delta R}{V_{\max}f_{c}T^{2}}}.$$

Suppose that *S* denotes the number of grids divided in the range from ${∥\alpha ∥}_{\min}$ to ${∥\alpha ∥}_{\max}$, and the value of α in the *i*th grid is:

(27) $$\alpha_{i}=\pm j\left({\left\|\alpha \right\|}_{\min}+\frac{{\left\|\alpha \right\|}_{\max}-{\left\|\alpha \right\|}_{\min}}{S-1}\left(i-1\right)\right),$$

where *I* = 1,…, *S*.

Considering {α_i_} as the set of all the possible values of α in *S* grids, and for each element in {α_i_}, we have the corresponding SDFnT matrix $G^{\alpha_{i}}$, and the range-Doppler radar image $Z^{\alpha_{i}}$ by using Algorithm 1.

We define the optimal value of α as the one that maximizes the peak value of ${\{Z}^{\alpha_{i}}\}$,

(28) $$\alpha_{\mathrm{opt}}=\arg\underset{\alpha_{i}}{\max}\left\{{\left\|Z^{\alpha_{i}}\right\|}_{\mathrm{peak}}\right\},$$

where $Z^{\alpha_{i}}$ denotes the peak value of the radar image $Z^{\alpha_{i}}$.

**Figure 1:**
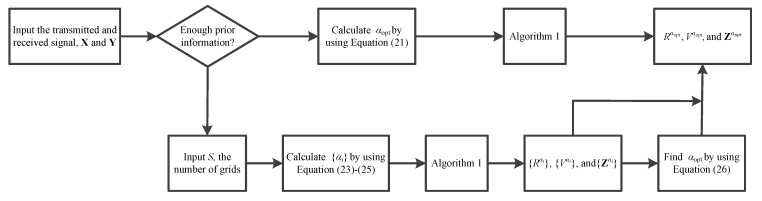
Flow chart of the proposed scheme.

Generally speaking, if there is enough prior information, we can calculate the optimal scale factor using Equation (21) and perform the proposed Algorithm 1 for range and velocity estimations. If there is not sufficient prior information for Equation (21), we can divide the range of possible α values into several grids, calculate the radar image for each grid value using Algorithm 1, search for the radar image with the maximum peak, and choose the value of α corresponding to it as the optimal scale factor. In conclusion, the signal processing flow chart of the proposed SDFnT ICI-free parameter estimation scheme is shown in Figure 1.

When there is insufficient prior knowledge, the accuracy of the optimal *α* appears to be dependent on the number of grids used in the searching method. Since the number of grids is preferred to be as minimal as possible to lower the computational cost, it could lead to a discrepancy between the estimated value of α and the true optimal value of *α*. Moreover, even with sufficient prior information, the calculated value of α is still susceptible to the influence of the estimation accuracy of the range and velocity while using Equation (21). In general, the value of the α employed in our proposed method is susceptible to errors between the true optimal value of α; therefore, in the next section of simulations and discussions, in addition to verifying the superiority of the proposed SDFnT-based ICI-free parameter estimation method, we will also investigate how the error in α affects the performance of the proposed algorithm.

## 4. Simulation Results

In this section, to demonstrate the performance of the proposed SDFnT-based method, we consider an OFDM radar system with a carrier frequency of 35 GHz and a subcarrier spacing of 100 kHz. The number of OFDM subcarriers is *N* = 256, and the number of symbols is *M* = 64. In this setting, the maximum range, range resolution, maximum velocity, and velocity resolution are *R*_max_ = 1500 m, ∆*R* = 5.86 m, *V*_max_ = 428.57 m/s, and Δ*V* = 6.70 m/s, respectively. To investigate the ICI mitigation effect of the proposed method, targets in high-velocity scenarios that lead to strong ICI effects are simulated. The conventional 2D-FFT method [26], the most widely used OFDM radar signal processing approach, is selected for comparison. Different experiments with single and multiple targets are carried out in the following subsections. In order to evaluate the robustness of our proposed method, we first present the impact of grid number on radar parameter estimation, followed by discussions of the impact of scale factor error in different scenarios on radar performance.

### 4.1. Impacts of Grid Number on Radar Performance

In this subsection, the effects of grid number on radar performance are simulated. The maximum and minimum values of the scale factor can be calculated as ${∥\alpha ∥}_{\min}=1$ and ${∥\alpha ∥}_{\max}=128$ by using Equations (23) and (24). The number of grids is set at 5, 10, and 20 to demonstrate the effect of different grid numbers on the performance of radar parameter estimation.

#### 4.1.1. Radar Performance in Terms of SNR

**Figure 2:**
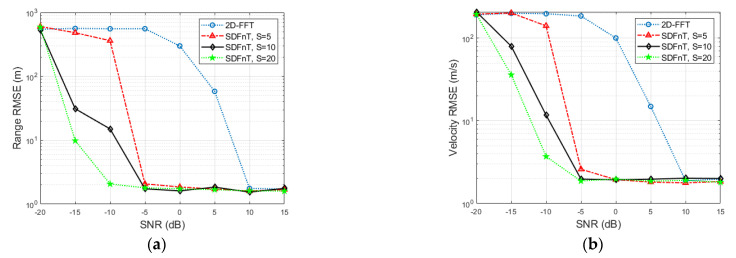
Estimation accuracy comparison in terms of SNR (**a**) RMSE for the range estimation (**b**) RMSE for the velocity estimation.

Consider a single target with a range of *R* = 400 m and a velocity of *V* = 360 m/s, which approaches the maximum velocity and leads to a severe ICI effect. Additionally, the SNR ranges from −20 to 15 dB with an interval of 5dB. To average the estimation results of our proposed algorithm and the conventional 2D-FFT method, 100 Monte Carlo simulations are carried out. For performance comparison, the root mean square error (RMSE) is calculated as

(29) $$\mathrm{RMSE}=\sqrt{\frac{{\left\|p_{\mathrm{est}}-p_{\mathrm{real}}\right\|}^{2}}{K}},$$

where *p*_est_ and *p*_real_ represent the estimated and true values, respectively. The RMSEs of the range and velocity estimates in terms of SNR are shown in Figure 2, where G represents the number of grids and SDFnT and 2D-FFT represent the proposed SDFnT-based ICI elimination algorithm summarized in Algorithm 1 and the conventional DFT technique in [26]. As expected, even in the high-velocity application, the proposed SDFnT method consistently achieves high accuracy in range and velocity estimations in the majority of the considered SNR region (≥−5 dB for *S* = 5 and 10; ≥−10 dB for *S* = 20). On the contrary, the performance of the 2D-FFT method deteriorates dramatically and fails to obtain range and velocity estimates when the SNR decreases. Moreover, as the number of grids increases, the size of the grids and the error of α decrease, and the proposed SDFnT-based method performs better at the expense of processing time.

#### 4.1.2. Radar Performance in Terms of Velocity

**Figure 3:**
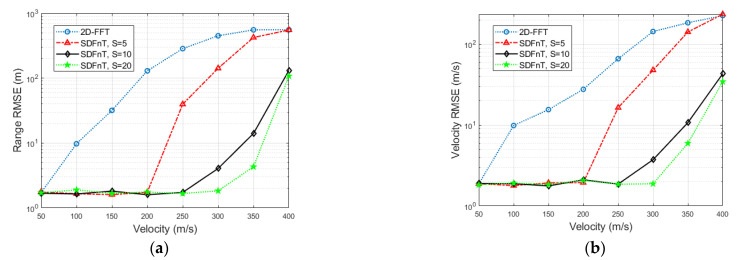
Estimation accuracy comparison in terms of velocity (**a**) RMSE for the range estimation (**b**) RMSE for the velocity estimation.

In order to analyze the accuracy of the proposed method with respect to velocity with poor signal quality, the velocity is set to vary from 50 to 400 m/s with an interval of 50 m/s, *R* is 400 m, and the SNR is set to −10 dB. After 100 Monte Carlo simulations, the RMSEs of the target parameters are shown in Figure 3. It is evident that the proposed SDFnT-based method outperforms the conventional 2D-FFT method within the considered velocity region, regardless of the grid number. Moreover, the scheme with fewer grids has higher RMSEs and is more sensitive to velocity increments for the same velocity condition. This is because, as the number of grids decreases, the error due to the scale factor increases, making it more difficult to eliminate the ICI-induced Doppler shift. In addition, the performance of the scheme with the grid numbers *S* = 10 and *S* = 20 is comparable, whereas the number of iterations is doubled. As a result, the scheme with the grid number *S* = 10 is preferable when system resources are limited.

In general, the proposed SDFnT-based technique consistently has outstanding range and velocity estimation performance at all signal qualities and target velocities regardless of the grid number for searching, which indicates the effectiveness of the ICI mitigation algorithm and its robustness against the grid number.

### 4.2. Single Target Simulations for Radar

In this section, radar performances for a single target in high-mobility applications are investigated. In order to reduce the computational cost, we assume there is sufficient prior information to calculate the optimal scale factor by using Equation (21) rather than the searching method. Furthermore, because errors in the range and velocity estimations are unavoidable (for example, in a tracking radar, we use the estimated parameters from the previous estimation to calculate the α_opt_ for the current estimation), the resulting α_opt_ may differ from the true optimal scale factor. As a result, in the following discussion of radar performance in terms of SNR and velocity, Δα, an error in α, is introduced as a parameter of interest. The Δα is set to be 0, 10%, 20%, and 40%, resulting in the α we use being α_opt_, 1.1 × α_opt_, 1.2 × α_opt_, and 1.4 × α_opt_, respectively.

#### 4.2.1. Range-Velocity Image

**Figure 4:**
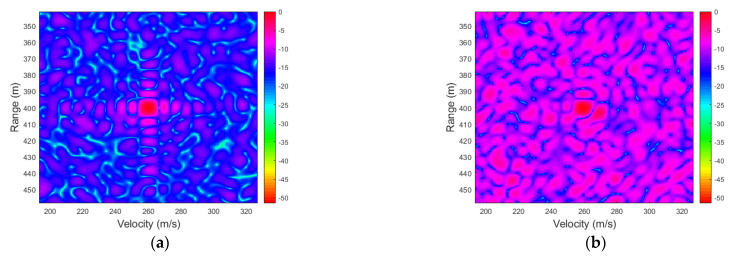
Normalized range-velocity image of a one-point target with *R* = 400 m and *V* = 260 m/s, level in dB. (**a**) SDFnT-based method. (**b**) 2D-FFT method.

Consider a single target with a range of *R* = 400 m moving at a high speed of *V* = 260 m/s, and the signal-to-noise ratio (SNR) is 20 dB. Figure 4 shows the normalized range-velocity images of the proposed SDFnT-based method with the optimal scale factor and the conventional 2D-FFT method. It can be seen that the proposed algorithm mitigates the ICI effects and accurately obtains the range and velocity of a high-speed target. In contrast, the ICI effects deteriorate the performance of the 2D-FFT method and prevent it from producing a clear range-velocity image.

The range-velocity image depicts the superior ICI elimination capability of our proposed technique for single targets with high velocity. However, despite appearing drowned in noise, the peak of the 2D-FFT radar image can still be found and solved for the correct range and velocity estimations. As a result, RMSE simulations are needed to evaluate the performance of the proposed technique in high-mobility radar for a single target.

#### 4.2.2. Radar Performance in Terms of SNR

**Figure 5:**
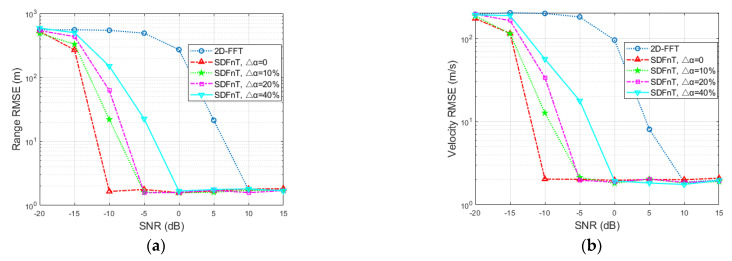
Single-target results in terms of SNR (**a**) RMSE for the range estimation (**b**) RMSE for the velocity estimation.

Figure 5 shows the RMSEs of the range and velocity estimates in terms of SNR after 100 Monte Carlo simulations. The velocity for simulation is set to 400 m/s, which approaches the maximum unambiguous velocity, to demonstrate the effectiveness of the ICI mitigation algorithm in high mobility scenarios. As can be observed, when the SNR decreases, the 2D-FFT method performs dramatically worse, whereas the SDFnT-based method can still estimate the high-speed target’s range and velocity accurately in low-SNR scenarios. Additionally, when the SNR becomes fairly low, our proposed method gradually fails, and the schemes with larger Δα are more sensitive to SNR reduction. In general, the proposed method consistently outperforms the conventional 2D-FFT method within the SNR range of interest and is resistant to scale factor error.

#### 4.2.3. Radar Performance in Terms of Velocity

**Figure 6:**
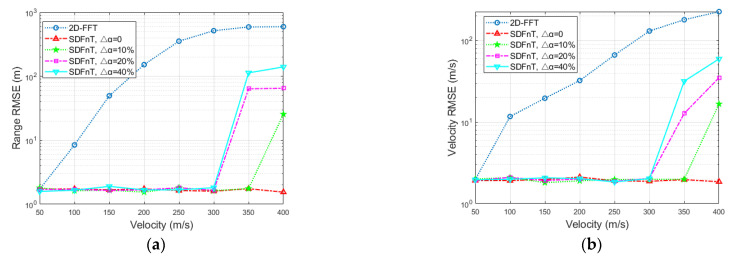
Single-target results in terms of velocity (**a**) RMSE for the range estimation (**b**) RMSE for the velocity estimation.

Figure 6 shows the RMSEs of range and velocity of a single target as a function of velocity for SNR = −15 dB after 100 Monte Carlo simulations. The velocity varies from 50 m/s to 400 m/s with an interval of 50 m/s. As evident in the figure, for the majority of the velocity range taken into consideration, the velocity increment has little effect on our proposed method but a substantial impact on the conventional method. Additionally, as the velocity approaches the maximum unambiguous velocity, the schemes with bigger Δα start to exhibit estimation errors, despite remaining superior to the conventional ones. In contrast, the schemes with smaller Δα constantly show excellent estimation performance.

Generally speaking, within the considered velocity range, the proposed SDFnT-based method significantly outperforms the traditional 2D-FFT method in terms of estimation performance, and as expected, exhibits strong robustness against the scale factor error.

### 4.3. Multiple Target Simulations for Radar

In this section, scenarios involving multiple targets are simulated. According to Equation (21), varied targets are related to different α because of their different speeds and distances. The scale factor α employed in multi-target detection is defined as the average of the targets’ own scale factors. The SNR ranges from −20 to 15 dB with a 5 dB interval, and the number of multiple targets is set to 3. If these three targets are clustered in the radar image, the average α should be almost the same as each α value. However, if the three targets are scattered in the radar image, the average value of α may be quite different from each α, necessitating a full discussion. As a result, the parameter estimation performances for clustered and scattered targets are investigated separately in the following discussion.

#### 4.3.1. Range-Velocity Image

**Figure 7:**
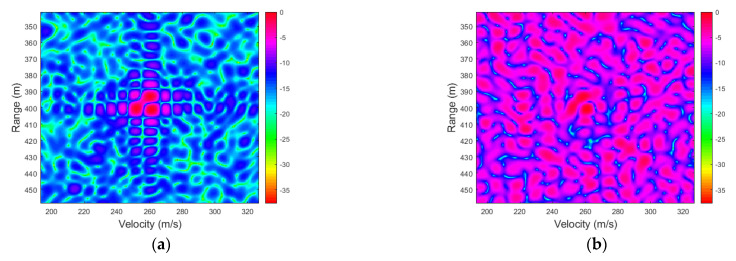
Normalized range-velocity image of three clustered targets with *R* = [400 m, 400 m, and 394 m] and *V* = [260 m/s, 253 m/s, and 260 m/s], level in dB (**a**) Using the SDFnT-based method (**b**) Using the 2D-FFT method.

Consider three targets with ranges of *R* = [400 m, 400 m, and 394 m] and moving at high speeds of *V* = [260 m/s, 253 m/s, and 260 m/s], respectively. The spacings between the targets are designed to be slightly greater than Δ*R* and Δ*V* to ensure that the simulated targets are clustered but not fused together in the radar image. The signal-to-noise ratio (SNR) is set to 20 dB. Figure 7 shows the normalized range-velocity images of the proposed SDFnT-based method with the optimal scale factor and the conventional 2D-FFT method. It can be seen that the proposed algorithm produces a clear range-velocity image, whereas the 2D-FFT method is severely affected by ICI effects and can hardly provide an accurate estimation of multiple targets since they are fused together in the resulting range-velocity image.

#### 4.3.2. Radar Performance for Clustered Targets in Terms of SNR

This subsection evaluates the performance of our proposed SDFnT-based method for high-speed clustered multiple targets in terms of SNR. The three targets are set separately at ranges of *R* = [400 m, 400 m, and 394 m] and move with speeds of *V* = [360 m/s, 353 m/s, and 360 m/s].

**Figure 8:**
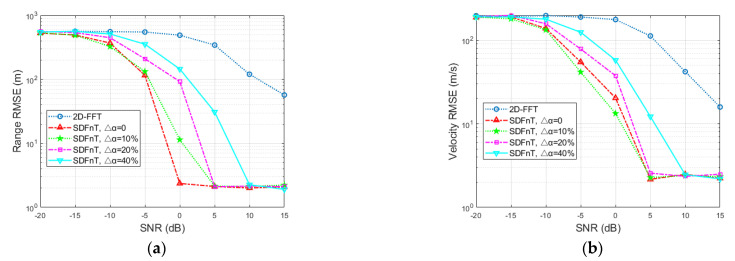
Clustered multi-target results in terms of SNR (**a**) RMSE for the range estimation (**b**) RMSE for the velocity estimation.

As observed in Figure 8, the proposed method consistently outperforms the conventional 2D-FFT method for the detection of clustered targets within the SNR range of interest and is robust to scale factor error. Furthermore, comparing Figure 5 and Figure 8 reveals that both the proposed and conventional methods perform slightly regressive when the applied targets are clustered rather than as single ones, particularly the conventional method, which is unable to accurately estimate the velocity and distance of the target even with SNR = 15 dB. This is due to the fact that all three targets are moving at high speeds, and the resulting ICI effect leads to the fusion of clustered targets on the radar map.

#### 4.3.3. Radar Performance for Scattered Targets in Terms of SNR

This subsection evaluates the performance of our proposed SDFnT-based method for high-speed clustered multiple targets. The three targets are set at ranges of *R* = [400 m, 200 m, and 400 m] and move with speeds of *V* = [360 m/s, 360 m/s, and 180 m/s]. The targets are designed to be very far apart in order to clearly demonstrate the performance of the proposed method when applied to dispersed targets with high velocities.

**Figure 9:**
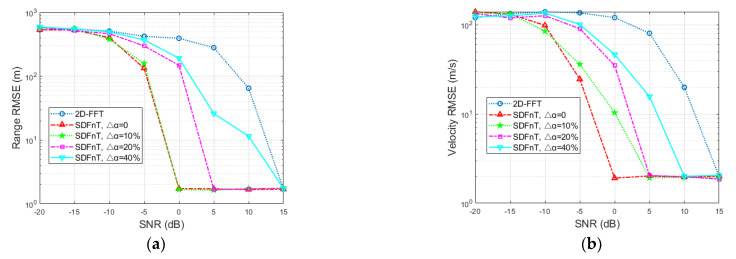
Scattered multi-target results in terms of SNR (**a**) RMSE for the range estimation (**b**) RMSE for the velocity estimation.

As illustrated in Figure 9, for clustered target detection, the proposed method consistently outperforms the conventional 2D-FFT method within the considered SNR range while being resistant to scale factor error. A comparison of Figure 8 and Figure 9 demonstrates that the performance of the SDFnT method when applied to scattered multiple targets with great distance is slightly worse than that of clustered multiple targets but still superior to the 2D-FFT method as a result of the average α used in the SDFnT method invariably having a larger error with the individual α values. Future applications of the suggested method for ICI mitigation may investigate employing mutually independent αs to estimate each target’s parameters.

## 5. Conclusions

In this paper, a novel SDFnT-based ICI mitigation method is proposed for OFDM radar systems in high-mobility scenarios. By transforming the signals into the scale Fresnel domain with an optimum scale factor, we suppress the non-negligible ICI effects caused by high-mobility targets and obtain the ICI-decoupled joint range and Doppler estimations. Simulation results have shown that the proposed approach has high robustness against the scale factor error and significantly outperforms the traditional 2D-FFT method for single and multiple targets. Since the SDFnT is compatible with DFT, our proposed method not only has a lower computation cost than the existing ICI mitigation methods but can also be directly implemented in the existing OFDM radar systems. Furthermore, the SDFnT and the ICI mitigation based on it could be applied for future multicarrier JRC waveform design and parameter estimation in high-mobility scenarios.

## Data Availability

Not applicable.
